# Impact of the Affordable Care Act on Stem Cell Transplantation

**DOI:** 10.1007/s11899-013-0191-0

**Published:** 2014-01-08

**Authors:** Stephanie Farnia, Alicia Gedan, Michael Boo

**Affiliations:** 1 Payor Policy, National Marrow Donor Program, 3001 Broadway Street, NE Suite 100, Minneapolis, MN 55413 USA; 2 Payor Policy & Legislative Relations, National Marrow Donor Program, Minneapolis, MN USA; 3 Administration-Business Development, National Marrow Donor Program, Minneapolis, MN USA

**Keywords:** Stem cell transplantation, Affordable care act, Essential health benefits, Health insurance, Health reform, Blood and marrow transplant, Bone marrow transplant, Reimbursement

## Abstract

The Patient Protection and Affordable Care Act, signed into law in 2010, will have a wide-reaching impact on the health care system in the United States when it is fully implemented in 2014. Patients will see increased access to care coupled with new insurance coverage protections as well as a minimum set of benefits mandated in each state known as essential health benefits. Providers are likely to see new forms of payment reform, particularly in the Medicare program, and narrower commercial provider networks. In addition, the composition of the health insurance market will broaden with the introduction of health insurance exchanges and expanded Medicaid populations in many states. Furthermore, the Patient Protection and Affordable Care Act calls for quality initiatives such as comparative effectiveness research to increase effective, appropriate and high-value care. This paper will review the main provisions of the Patient Protection and Affordable Care Act with specific attention to their impact on the field of Stem Cell Transplantation.

## Introduction

The Patient Protection and Affordable Care Act [1], known as the ACA, was signed into law on 23 March 2010. The law is intended to increase access to health care while reducing the overall cost of health care. On the whole, patients who have had or who will need a stem cell transplant should benefit from expanded access to affordable insurance options and the removal of long-standing benefit and coverage restrictions as provided under the ACA. However, there remains significant room within the law that raises other concerns regarding access that will need to be monitored for potential impact to patients and providers. In this article, we will explore specific sections of the ACA and their potential impact on patients and providers. We will suggest ways in which the provider community may want to respond to the new challenges and opportunities presented by the law in order to best serve themselves and their patients.

### Implementation

The provisions of the ACA are phased in over a period of time to allow stakeholders time to prepare. While a number of provisions have already been implemented, those changes that significantly expand access to health care or modify insurance coverage requirements go into effect on 1 January 2014. These include, among many others, expanded Medicaid coverage, access to insurance marketplaces through state and federal healthcare insurance exchanges and the elimination of insurance provisions such as annual and lifetime maximum benefit dollar amount limits.

Efforts to reduce the cost of healthcare are also being phased in. These initiatives create incentives for healthcare payors (health plans, employers and other purchasers) and providers to pursue quality improvement strategies together. The goal is to achieve efficiencies in the delivery of healthcare by reducing administrative costs, mandating the use of quality measure reporting, focusing on delivering appropriate care and by using comparative effectiveness studies to better define and guide patients to the most appropriate care.

Physicians and the patients they treat are anxious to understand the impact of this legislation as it is being implemented [2]. Some components, such as the Medicaid expansion, are fairly well established, but as for the definition of benefits and provider networks available through the exchanges, they are still coming into focus. The exchanges opened to consumers on 1 October 2013, and the offerings are being analyzed by many interested parties, including the National Marrow Donor Program (NMDP).

### Stem Cell Transplant: Unique Areas of Concern

Stem Cell Transplantation (SCT) is a resource-intensive therapy for critically ill patients with hematologic malignancies and other illnesses. These patients need intensive, and frequently expensive, treatment regimens both prior to and after transplant. Patients who receive a SCT continue to need access to specialized providers and treatment for the remainder of their lives. The severity of illness in patients in need of a transplant, as well as the transplant process and recovery period, almost always leads to a period of time in which a patient is absent from the workplace. This often results in marked decrease in income during a period of increased medical expenditures, as well as potentially jeopardizing the individual’s ability to maintain his or her health insurance coverage under typical insurances plans in existence today. For years after the SCT, patients routinely face high out-of-pocket spending because of on-going follow-up care, lab testing and prescription drugs.

Prior to the enactment and implementations of the ACA, SCT patients seeking new insurance coverage in the individual marketplace historically faced the potential of a lack of insurers willing to insure them or limited benefit insurance plans with prohibitively high premiums and pre-existing condition exclusions of any costs related to the transplant.

The forthcoming changes to the health care and insurance systems will positively impact SCT patients more quickly and to a greater degree than an average health care consumer. We will highlight how certain provisions will impact current or former SCT patients throughout this discussion.

## Expanding Access to Healthcare

A primary goal of the ACA is to reduce the number of uninsured Americans. The ACA increases access to health insurance through three primary avenues. First, it requires existing insurance coverage, such as through employer provided health plans, to meet certain standards of coverage and benefits. Second, it creates a mechanism, known as the healthcare insurance exchanges, through which individuals and small groups can compare and purchase health insurance policies. Third, in states that elected to participate, it expands the individual and family income limits that determine eligibility for participation in a Medicaid program.

The drafters of the ACA understood that simply organizing a market for insurance products would not likely compel all potential enrollees to participate. Those without any known or current health concerns may decide to wait until an issue occurs and then subscribe. This would create adverse selection where only those who have a known need for health insurance, outside of preventive care, purchase it. This defeats the goal of creating market based affordable health insurance as there are no low-cost members to off-set the claims expenses of high-cost members. Thus, the law provides that individuals not otherwise covered under Medicare, Medicaid, or employer provided health plans must purchase insurance coverage or face a financial penalty. This assures the broadest base of consumers against which the payors can price the products. Further, the law limits segmentation of the market for pricing purposes. Premium assistance subsidies based upon consumer need as measured by individual or family income are also provided under the ACA to assure all have the means to acquire insurance through the exchanges.

### Applicability of New Requirements

As noted, one of the goals of the ACA is to assure that all individuals have insurance coverage. The law attempts to preserve the existing private based insurance system that covers most Americans, while bringing some consistency to the benefits provided across all insurance products. However, the law does not mandate complete uniformity, and leaves room for changes over time in existing plans to move toward a more common benefit set.

The private insurance market is defined by three main categories through which consumers obtain coverage. The first category is insurance provided to individual purchasers who purchase the insurance as a single person or a person and his/her dependents that purchase a policy directly from an insurer. The second category is insurance acquired through group purchasing, typically provided by employers. Groups that are fully insured pay a monthly premium to a health insurance company and the insurance company assumes all risk – i.e., all responsibility for paying claims incurred by that group, regardless if the cost total is more than the premiums paid. The final category is coverage provided directly by an employer who takes all risk in providing coverage, referred to as self-insured or self-funded plans. Commonly, employers with self-insured plans protect against financial risk through another form of financial insurance, known as stop-loss insurance, to protect against unforeseen individual or aggregate costs above an expected dollar amount. Self-insured groups contract with claims administrators or health plans to handle the administration of their health benefits and give them access to a contracted provider network.

The ACA allows for the continuation of certain plans currently in existence, known as “grandfathered” plans even though they may not follow all of the requirements of the ACA. Grandfathered plans are those that were purchased prior to the law’s signing in 2010 and that have not made any changes of specific types that dictate maintenance of this status. Table 1 shows which ACA requirements apply to grandfathered plans. The number of grandfathered plans is expected to fall substantially over the next few years due to the nature of the changes that most plans will need to make during this time [3].

**Table 1:** Grandfathered Plans Description

Applies to Grandfathered Plans	Does Not Apply to Grandfathered Plans
Removal of Lifetime Limits	Out-of-Pocket Maximum Limits
Prohibition of Coverage Rescission	Coverage of Clinical Trial Routine Costs
Coverage of Dependents up to age 26	Coverage of Preventive Care w/o Cost-Sharing
Prohibition of Pre-Existing Conditions	Essential Health Benefits
Waiting Period Limit of 90 Days	Compliance with a Metal Tier Category

Due to the various mechanisms through which insurance can be provided to employees and to the grandfathered/non-grandfathered status issue, it will be very important that all transplant programs review the individual benefits for each patient. Very few assumptions can be made about the insurance provision changes outlined in the following section.

### Changes to Insurance Coverage and Benefit Provisions

The ACA assures access to health insurance in a number of ways, particularly for those purchasing coverage through the individual marketplace. The ACA creates “guaranteed issue” requirements– meaning that anyone eligible for insurance cannot be turned away – and prevents insurers from rescinding coverage from someone when they are diagnosed with an illness or condition. Lifetime dollar limits on total paid benefits are no longer allowed after 1 January 2014. Annual dollar limits are allowed only in a much more restricted manner, specifically for services not covered by the definition of the Essential Health Benefits, discussed below, or those provided by a grandfathered plan.

The ACA also removes pre-existing condition exclusions. This means that patients who have had a hematologic malignancy or other significant health condition in the past cannot be excluded from coverage or held responsible for medical costs associated with those conditions in the future. This will be particularly helpful to individuals who were pediatric SCT patients and are now attempting to secure their own individual coverage as an adult. Stem cell donors will also benefit, because a history of donation cannot be used to deny them purchasing an individual plan. Finally, since 2011, the ACA required that health plans allow dependent children to stay on their parents’ individual or group health plan until they are 26 years old. This is very useful for adolescent and young adult SCT patients who want to maintain consistent coverage and benefits at their original transplant center for an extended period.

Another important requirement that will benefit SCT patients is the right that insured members have to an external, independent review of a benefit or care denial by a health plan. This provision has been in place since 2012 and mandates that health insurance companies inform patients of the external review process and whether a patient’s state has a Consumer Assistance Program that could be of help during the appeal review process. The expanded review process may be helpful for patients pursuing transplant since many SCT patients face denials on a variety of authorization requests, such as drug regimens, follow-up care items, a second transplant or additional cell infusion, or a transplant for a relatively new treatment indication. An area of concern for this provision is that the credentials of the external reviewer are not spelled out. When seeking an external review, patients and physicians should consider making a specific request for evaluation by a transplant physician or hematology/oncology specialist.

### Essential Health Benefits

The ACA mandates that health plans available to consumers offer a comprehensive set of benefits, referred to as the Essential Health Benefits (EHB). The Federal regulations require ten areas of care to be included in all non-grandfathered health plans. The ten areas are (1) ambulatory patient services, (2) emergency services, (3) hospitalization, (4) maternity and newborn care, (5) mental health, (6) prescription drugs, (7) rehabilitative (skill recovery) and habilitative (skill acquisition) services, (8) laboratory services, (9) preventative and wellness services and (10) pediatric services. While there is not a specific mention of SCT as an EHB at the Federal level, the components of the transplant process all fall into covered categories.

**Fig. 1:**
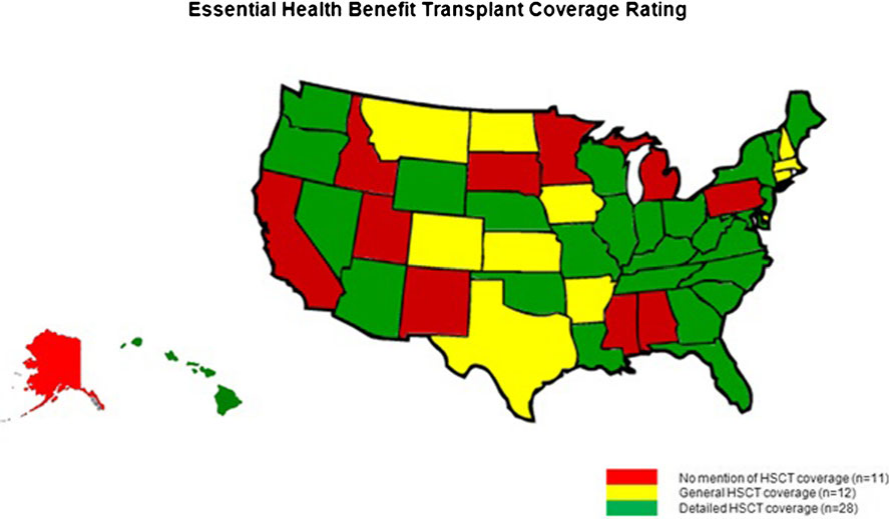
Essential Health Benefit Benchmark Plans SCT Analysis

In developing the benefit requirements in state exchanges, states are not limited to the EHB set detailed by Health and Human Services (HHS), and many states have provided additional EHB requirements for plans being offered for purchase. In 2013, the NMDP conducted an analysis of every state’s EHB benchmark plan – the plan being used as a model for others being offered through the exchange - looking for SCT benefits. Twenty-eight states have specifically mentioned various components of SCT benefits in their EHB benchmark plan documents, providing additional reassurance for patients and providers in those areas (Fig. 1). However, transplant programs should note that there were no uniform modifications or improvements made to transplant-specific benefits. Donor search, cell procurement, and travel and lodging benefits will continue to be limited in most areas and problematic for patients beginning the transplant process.

### Clinical Trials Coverage

Clinical trials coverage is a very important category to SCT patients and their providers. Under the ACA, coverage for routine costs associated qualifying patients within an approved clinical trial will be required beginning in January 2014. Routine costs are defined as all aspects of care outside of the investigational drug, item or procedure itself. Clinical trials must be approved or sponsored by the National Institutes of Health (NIH), the Center for Disease Control and Prevention (CDC), The Agency for Healthcare Research and Quality (AHRQ), Centers for Medicare & Medicaid Services (CMS). Trials may be any phase (I-IV) and must be conducted in relation to the prevention, detection, or treatment of cancer or other life-threatening disease or condition. The Blood and Marrow Transplant Clinical Trials Network (BMT CTN) is funded by two divisions at the NIH and, thus, its trials should qualify for routine cost coverage. Transplant programs within a National Cancer Institute (NCI) Designated Cancer Center may also face reduced barriers to patient clinical trial participation based on this provision. In cases where transplant itself is the investigational item of interest – i.e., the transplant is being investigated for a new indication - payment for the cells and hospital stay may not be covered. The nuance of implementing this provision will need to be monitored for expected impact on SCT research. Grandfathered plans do not have to recognize the ACA requirement for clinical trials coverage, so transplant centers may want to build an additional coverage verification step into their financial planning process. However, state laws may separately require clinical trial coverage under certain conditions.

Transplant centers will need to provide clear communication to payors regarding the qualification of the trial, the eligibility of the patient, and the portions of the treatment plan that are routine or investigational.

### Health Insurance Marketplaces – The Exchanges

The creation of the state and federal online marketplaces, known as Health Insurance Exchanges (exchanges) [1], is intended to assure access to insurance for individuals who do not purchase insurance directly from an insurer or who have coverage through their employer. The exchanges simplify the process of identifying coverage options, assist in determining potential Medicaid eligibility and assess an individual’s premium subsidy qualification. The exchanges will provide consumers with a menu of private insurance options available to them in their state categorized by insurer, premium, out-of-pocket costs, deductible and provider network. The number of plans offered and the composition of insurers on the exchanges will vary greatly from state to state. Sixteen states and the District of Columbia will be running their own exchanges while twenty-seven have defaulted to a federally facilitated exchange model. The remaining seven states will partner with the federal government in running their exchange with the goal of creating a state run exchange in the future.

In 2014, thirty-one states will also offer the option to enroll in one of the plans available to Federal Employees known as the Multi State Plan Program run through the federal Office of Personnel Management. The benefits these plans offer transplant patients will become increasingly important both as a de facto benchmark and due to their direct applicability to a large number of enrollees.

In order to make the plan offerings easily comparable for consumers, all plans offered through an exchange have to offer benefits that align with one of the “metal tiers” – bronze, silver, gold or platinum. These metal categories align with different estimated percentages (ranging from 60 % to 90 %) of expected annual average costs of services incurred within the EHB categories that will be paid for by the plans. Bronze plans will have more affordable premiums, with more out-of-pocket spending through variables such as deductibles, co-pays and co-insurance. Platinum plans will likely have higher premiums and less variable cost. Gold and platinum plans may also have more benefits outside of the EHB categories, such as travel and lodging expenses during a transplant. SCT patients should carefully investigate plan options and may consider incurring a more expensive fixed monthly premium to minimize fluctuation in out-of-pocket spending during a time of limited family income. All of the plans face the same limits on out-of-pocket maximum limits: $6,350 for an individual or $12,700 for families [4].

The exchanges will be replacing state-run high-risk pools (individual insurance pools for those with health conditions who cannot access coverage otherwise) in most states. Many SCT patients have participated in high-risk pools in the past and may need assistance from their transplant center in evaluating their insurance options through the exchanges. Transplant programs may want to review the plans available in their local exchange for transplant-specific benefits and create a list of plans with benefits that are supportive of the transplant process [5].

### Provider Networks in the Exchanges

Health insurance companies offering plans through the exchanges will likely have limited provider networks in order to keep the premiums affordable and competitive. These limitations in network size have the potential to create delays and obstacles for patients trying to make appointments for the evaluation of initial symptoms or with specialists for treatment planning and follow-up care. An even greater concern is that patients may sign on to a plan with a network that does not offer all transplant options. For instance, in Minnesota at the time of this writing, only nine of the thirteen plans available through the state exchange have an allogeneic stem cell transplant center in their provider network [6]. Whether patients will be allowed access to out-of-network providers in cases of specific transplant type or disease indication remains to be seen. Transplant programs should research which Exchange plans their facility is participating in and be prepared to communicate this to current and future patients.

### Medicaid Expansion

The ACA provided that all states must expand coverage under Medicaid to individuals up to 133 % of the Federal Poverty Level (FPL) and provided federal funding to cover the cost of increased coverage. However, in 2012, the United States Supreme Court declared that this requirement was unconstitutional and that each state had the right to decide whether or not to implement this provision of the ACA. As a result, the extent of Medicaid coverage is to be determined on a state-by-state basis. As of November 2013, twenty-five states have elected to expand coverage and receive the associated federal funding. Five other states are still considering expanding their Medicaid programs in an effort to reduce their state insurance coverage gaps. There is no deadline for expanding Medicaid programs and many more states are expected to expand their programs in the near future. Medicaid expansion is expected to increase the number of enrollees from 48.3 million to 66.4 million [7]. States may determine if they will add the newly eligible low-income adults to their current adult benefit package or if they will provide an alternative benefit package to this group. The benefit package made available to the expansion population is required to meet the EHB specifications as determined by the state benchmark plan.

Expanded Medicaid will have both positive and negative repercussions for patients and transplant programs. Increased access to coverage will mean more patients have SCT as a treatment option, but this expansion does not improve the quality of benefits or the reimbursement rates associated with state Medicaid plans. An increase in Medicaid patients with these less-than-ideal coverage provisions will mean an increased burden on already limited transplant center resources [8]. Transplant centers in states with problematic Medicaid coverage indications, benefits, or extremely low reimbursement levels may want to consult with government policy staff at their medical centers to develop an outreach plan to state health officials.

## Payment Reform

In addition to access, the other significant tenet of the ACA is an overall reduction in healthcare spending, particularly in the Medicare program. The applicable provisions include penalties for certain readmission types, stricter fraud and abuse regulations, and incentives for the adoption and use of quality measurements. Hospitals are facing additional payment changes through reductions to Disproportionate Share Hospital (DSH) payments, graduate medical education spending and reduced physician fee payments. These reductions are intended to encourage providers to identify and utilize efficient care practices while reducing unnecessary care [9].

Incentives are also provided to encourage the development of Accountable Care Organizations (ACOs) and the use of bundled payments. ACOs are designed to transfer responsibility for management of healthcare services to provider-based organizations that are provided funds to manage the healthcare needs of a population rather than payment for specific services. Bundled payments are designed to extend current payment methodologies, such as the Diagnosis Related Group (DRG) payments used in the Inpatient Prospective Payment System (IPPS), over a broader set of healthcare services around specific diagnoses. Under a bundled payment scheme, a single payment may include professional services, facility payments and aftercare arrangements. Groups of providers will be encouraged to work together to manage patients in a more efficient and cost-effective manner.

As is the case with many of the provisions of the ACA, the expected impact on transplant centers is uncertain. However, it will likely be significant since Medicare eligible patients are the fastest growing segment of allogeneic unrelated transplants [10]. Transplant centers will be well-served by preparing for new models of payment bundling, pay-for-quality programs and an increased focus on cost-effectiveness and value from all payor types. Transplant centers will be under pressure to document quality of care, measured by both specific elements of care provided to patients as well as outcomes, to avoid penalization or earn incentives. In addition, while transplant physicians and centers have historically worked closely together to manage patient care, the development of ACOs and bundled service arrangements may extend the network of providers for which the transplant physician and center will need to interact in the management of the patient and the time frame over which the assigned payment will extend. The transplant community needs to begin considering how to best collect and identify true costs of care delivery and how to communicate the value and cost-effectiveness of transplantation.

An increased focus on quality initiatives is expected to drive a decrease in spending by providing effective, appropriate and high-value care. The ACA established the Patient-Centered Outcomes Research Institute (PCORI) to identify research priorities and sponsor comparative effectiveness research on treatment options. Medicare will also be expanding its physician quality measure reporting program to additional specialties. SCT physicians interested in individual and team outcome assessment should begin to identify quality measures of potential interest.

## Conclusion

The Affordable Care Act is ambitious, broad-reaching and complex. There will be a significant amount of turmoil for several years after the implementation of the 2014 requirements. Physicians and transplant centers will likely need to assist patients with their navigation of the new Exchanges and networks in order to access the specialized care they need. Additional federal regulatory guidance is needed to clarify the more complex ACA provisions and their interactions with our health care system. Transplant centers should not assume that patients understand this new healthcare world or that the insurance benefits that providers were used to working with are still applicable. Keeping up-to-date on key factors of the ACA and how implementation is being handled by the states in the transplant centers referral area will be crucial to providing relevant and comprehensive patient support.
